# The effect of dobutamine stress on diastolic filling rates in obese subjects

**DOI:** 10.1186/1532-429X-11-S1-P141

**Published:** 2009-01-28

**Authors:** Oliver J Rider, Jane M Francis, Tammy Peggy, James P Byrne, Kieran Clarke, Stefan Neubauer

**Affiliations:** 1 University of Oxford, Oxford, UK; 2 Southampton General Hospital, Southampton, UK

**Keywords:** Dobutamine, Obese Subject, Diastolic Function, Filling Rate, Left Ventricular Filling

## Objective

Our aim was to determine the effect of catecholamine stress on left ventricular filling rates in obesity and compare this to normal weight subjects.

## Background

Obesity is associated with diastolic dysfunction at rest. However, it is unknown whether this is exacerbated during stress, i.e. whether the "relaxation reserve" of the heart is impaired in obesity. We aimed to determine the effect of simulated exercise on diastolic function in obese subjects without co-morbidity, and compared this to normal weight subjects.

## Methods

45 healthy subjects (28 obese, BMI 39.8 ± 8.0 &17 lean controls 21.6 ± 1.6) were enrolled into the study. All subjects were screened for identifiable cardiac risk factors and excluded if present. All subjects underwent cardiac MR imaging at 1.5 T for peak diastolic peak filling rate (PFR, derived from LV volume time curves, normalized to end-diastolic volume). 11 of the normal weight and 20 obese subjects underwent repeat assessment of diastolic function during dobutamine infusion.

## Results

**Figure 1:**
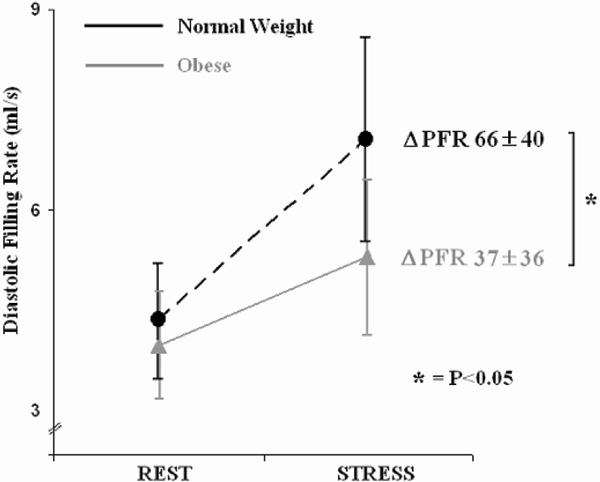
**The effect of dobutamine infusion on peak filling rates in obese and normal weight subjects**.

At rest, obesity was associated with a 20% reduction in LV peak filling rate (3.6 ± 0.8 vs 4.6 ± 1.0 ml/s, p = 0.01). As would be expected from the action of dobutamine, in normal weight subjects, with an increase in heart rate (HR) of 60% during stress (from 63 ± 9 to 99 ± 15 bpm) there was a 66% increase in maximal PFR; furthermore, HR and diastolic function showed a close linear correlation (R^2^ 0.86, p < 0.001). In contrast, in obese subjects, a 62% increase in HR (from 65 ± 9 to 105 ± 9) resulted in a significantly smaller, 37% (vs 66%, p < 0.05) increase in PFR, Figure 1. Furthermore, the correlation between HR and diastolic function was no longer significant (p = 0.24).

## Conclusion

Similar levels of stress result in significantly smaller increases in diastolic PFR in obese compared to lean controls. Thus, the "relaxation reserve" of the heart is impaired in obesity.

